# Effect of Strain Rate on Hydrogen Embrittlement of Ti6Al4V Alloy

**DOI:** 10.3390/ma17051100

**Published:** 2024-02-28

**Authors:** Tien-Dung Nguyen, Nooruddin Ansari, Keun Hyung Lee, Dong-Hyun Lee, Jun Hyun Han, Soo Yeol Lee

**Affiliations:** Department of Materials Science and Engineering, Chungnam National University, Daejeon 34134, Republic of Korea; dungnt.mse@gmail.com (T.-D.N.); nooruddin1992@gmail.com (N.A.); porary95@o.cnu.ac.kr (K.H.L.); dhlee@cnu.ac.kr (D.-H.L.); jhhan@cnu.ac.kr (J.H.H.)

**Keywords:** Ti6Al4V alloy, hydrogen embrittlement, strain rate, solute hydrogen

## Abstract

The phenomenon of hydrogen embrittlement (HE) in metals and alloys, which determines the performance of components in hydrogen environments, has recently been drawing considerable attention. This study explores the interplay between strain rates and solute hydrogen in inducing HE of Ti6Al4V alloy. For the hydrogen-charged sample, as the strain rate was decreased from 10^−2^/s to 10^−5^/s, the ductility decreased significantly, but the HE effect on mechanical strength was negligible. The low strain rate (LSR) conditions facilitated the development of high-angle grain boundaries, providing more pathways for hydrogen diffusion and accumulation. The presence of solute hydrogen intensified the formation of nano/micro-voids and intergranular cracking tendencies, with micro-crack occurrences observed exclusively in the LSR conditions. These factors expanded the brittle hydrogen-damaged region more deeply into the interior of the lattice. This, in turn, accelerated both crack initiation and intergranular crack propagation, finally resulting in a considerable HE effect and a reduction in ductility at the LSR. The current study underscores the influence of strain rate on HE, enhancing the predictability of longevity and improving the reliability of components operating in hydrogen-rich environments under various loading conditions.

## 1. Introduction

With high strength, low density, and good corrosion resistance, Ti6Al4V (Ti64) stands out among titanium alloys [1,2]. Ti64 has drawn significant attention in various industries, including the biomedical, manufacturing, maritime, and aerospace fields [3,4,5,6]. In the aerospace sector, Ti64 displays commendable performance under demanding conditions and stringent mechanical requirements. However, a significant environmental obstacle for Ti64 during operation is the occurrence of hydrogen embrittlement (HE) [7,8,9,10,11,12,13,14,15,16,17].

The HE of Ti64 is demonstrated by the distinctive failure mechanism characterized by crack initiation and propagation due to the accumulation of solute hydrogen or the presence of a hydride phase. Depending on the material and its microstructure, solute hydrogen can enhance both the dislocation density and the dislocation mobility in the stress field regions with a high concentration of hydrogen over a critical level [18,19,20]. Upon surpassing the hydrogen solubility limit, the formation of a brittle titanium hydride phase ensues, accompanied by volume expansion and lattice strains, as reported by Deconinck et al. [21]. Titanium hydrides play a pivotal role in accelerating mechanical degradation, leading to premature failure, particularly in hydrogen-rich environments [22]. Indeed, Shih et al. [23] demonstrated in Ti-4Al-6V, an α-alloy, that hydrogen-assisted fracture involves a combination of stress-induced hydride formation (SIHF) and hydrogen-enhanced localized plasticity (HELP). The majority of research on HE has been focused on the processes of hydrogen absorption and the subsequent behavior of absorbed hydrogen within the metals [7,8,9,10,11,12,13,14,15,16,17]. In practical applications, the longevity and reliability of components are paramount considerations. Therefore, it becomes imperative to thoroughly investigate the behavior of metals, particularly regarding HE, under a spectrum of loading conditions. However, the primary focus in prior research has been on comprehending HE within specific loading scenarios, potentially neglecting the influence of strain rate variations [10,11,16,17]. This narrow scope of research fails to address the comprehensive range of real-world conditions that such materials may encounter. The question arises as to how strain rates influence the occurrence of HE in Ti64.

It is known that the strain rate sensitivity of a material is influenced not only by its microstructure but also by the mechanism of deformation [18,24,25,26,27]. In the case of titanium alloys, numerous investigators have examined the correlation between microstructural evolution and strain rate in an air environment [28,29,30]. However, the sensitivity of the strain rate in the hydrogen environments is seldom reported. It is reasonable to anticipate that the presence of solute hydrogen could influence the strain rate sensitivity. This concept can be substantiated by the pivotal role determined by the strain rate in shaping the dynamics of dislocation-hydrogen competitive motion, in addition to the potential consequences on the evolution of dislocation patterns and cracking behavior [18,19,20]. Previous studies have reported that the susceptibility to HE increases with a decreasing strain rate [18,24,25,26,27]. However, a fundamental understanding of the intricate interplay between hydrogen and the microstructure under different strain rates remains elusive, necessitating further comprehensive investigation.

This study marks the first exploration into how solute hydrogen and strain rates collectively influence the deformation behavior of Ti64 at room temperature. The solute hydrogen was introduced into the Ti64 alloy, and subsequent uniaxial tensile tests with different strain rates were performed on both hydrogen-charged and uncharged samples. The current findings reveal the role of the strain rate on microstructural evolution, the contribution of solute hydrogen to hydrogen-assisted cracking behavior, and their combined effects on the HE. In contrast to samples tested at high strain rates (HSR), which experienced relatively short deformation times, low strain rate (LSR) tests resulted in more pronounced microstructural changes. This was evidenced by a significant increase in the length of high-angle grain boundaries (HAGBs). Consequently, the diffusion and accumulation of hydrogen were heightened in the LSR-tested samples. This led to a more severe manifestation of HE, as demonstrated by a substantial decrease in ductility. Therefore, this study not only supports material selection but also lays the groundwork for devising processing methods, creating favorable conditions for the development of Ti64 alloys optimized for superior performance in hydrogen-rich environments under various loading conditions.

## 2. Materials and Methods

A commercial Ti64 plate (Baoji Hongyi Titanium Industry, Baoji, China) with dimensions of 300 × 150 × 10 mm^3^ was used in this study. The material underwent treatment processes involving rolling at 950 °C for 2.5 h, followed by annealing at 750 °C for 2 h, according to ASTM B265 [31]. The chemical composition of the alloy is presented in Table 1.

Dog-bone-shaped plate-type tensile samples with gauge dimensions of 12.5 mm (length) × 6 mm (width) × 1 mm (thickness) were machined from the Ti64 plate. The profile surface was represented in a rolling direction–transverse direction (RD-TD) plane. The activation of solute hydrogen was achieved through the electrochemically hydrogen-charged method. The electrolyte employed in this process consists of a mixed solution containing 0.5 mol/L of H_2_SO_4_ and 3 g/L of NH_4_SCN (thiourea). Thiourea was deliberately introduced to enhance the absorption of atomic hydrogen into the materials, capitalizing on its inhibitory effect on the hydrogen recombination reaction that leads to the formation of molecular H_2_. Galvanostatic mode was carried out using a constant current density, specifically set at 1 mA/cm^2^. Additionally, this charging procedure continued for 24 h at room temperature. Tensile tests were conducted for both hydrogen-charged and uncharged samples, in which the loading direction was parallel to the rolling direction (RD) and strain rates ranged from 10^−2^ to 10^−5^/s. These strain rates were chosen with the purpose of promoting the HE with a simultaneous reduction in the mechanical parameters.

Microstructural characterization was performed using a scanning electron microscope (SEM) equipped with electron channeling contrast imaging (ECCI) and electron backscatter diffraction (EBSD; NordlysNano, Oxford, UK) to clarify the hydrogen-affected cracking behaviors. The samples were mechanically ground with a series of SiC papers up to a 2000# grid, polished by using 3 µm diamond suspension, and then subjected to a 30 min final polishing step using a solution containing 50 mL of 0.04 µm colloidal silica suspension, 10 mL of H_2_O_2_ (30%), and 5 mL of Kroll’s reagent for microstructural examinations. Microstructural deformation was evaluated by observing the microstructures of both the initial and fractured samples on the profile surfaces. The influence of solute hydrogen on the cracking behavior was performed through analyses of microstructural evolution, specifically focusing on the propagation of secondary cracks on the cross-section of the hydrogen-charged sample. Furthermore, the fracture surfaces of both hydrogen-charged and uncharged samples were examined to compare the fracture behavior under various strain rates.

## 3. Results and Discussion

**Figure 1:**
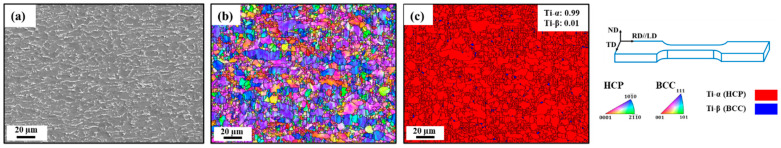
Initial microstructure of the uncharged sample on the RD-TD plane: (**a**) scanning electron microscope (SEM) image, (**b**) inverse pole figure (IPF) map, and (**c**) phase map from EBSD.

The initial microstructure of the uncharged sample on the RD-TD plane is shown in Figure 1. The SEM image and inverse pole figure (IPF) map together illustrate a distinctive pattern: larger grains exhibited elongation along the RD within a randomly distributed matrix of smaller grains (Figure 1a,b). This is likely a result of the recrystallization process occurring during post-rolling annealing. Further insight into the microstructural features of the initial sample is provided by the phase map in Figure 1c. The phase map indicates the coexistence of α and β phases within the microstructure and demonstrates that the β phase was predominantly situated at the boundaries of α-phase grains, constituting only 1% of the total fraction (Figure 1c).

**Figure 2:**
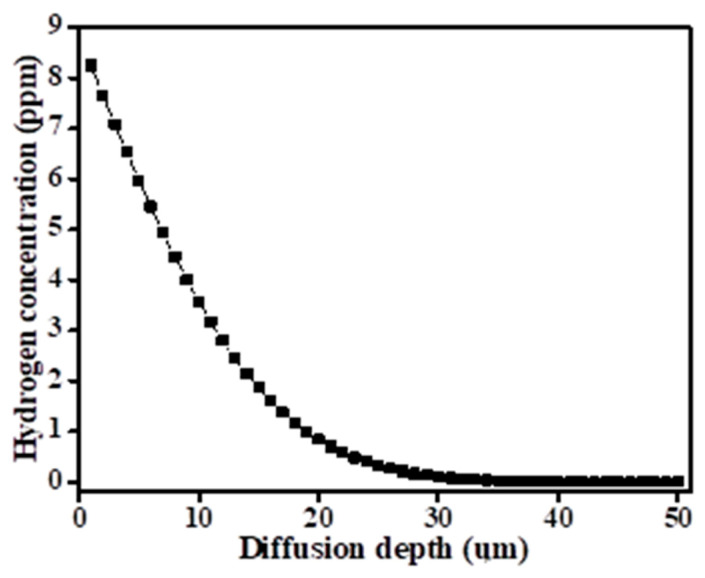
The theoretical estimation of the hydrogen diffusion profile on the hydrogen-charged sample for 24 h with a current density of 1 mA/cm^2^ at room temperature.

The hydrogen diffusion profile of Ti64 alloy after 24 h of charging at 1 mA/cm^2^ at room temperature, as depicted in Figure 2, was derived from theoretical calculations. Regarding the diffusion distance of hydrogen, the theoretical hydrogen diffusion profile in a semi-infinite plate, based on Fick’s second law and models by Crank [32], was estimated using Equation (1). Similarly, Demetriou et al. presented analogous findings using Equation (1) to characterize the hydrogen diffusion profile following electrochemical hydrogen charging for 945X (UNS N09945) [33], and Deconinck et al. presented the same for Ti64 alloy [21]. In the equation, *c(x, t)* is the hydrogen concentration at a specific location, *c*_0_ is the initial concentration, *c_s_* is the electrolyte concentration, *x* is the depth (µm), *D* is the diffusion coefficient (m^2^/s), and *t* is the time (s). The general diffusion coefficient of 10^−14^ m^2^/s was employed. Only hydrogen introduced through the charging was considered. From the production processes, it was assumed that the hydrogen remained after the operation was uniformly distributed over the specimen. Furthermore, the theoretical maximum hydrogen concentration near the exposed surface (approximately 8.3 ppm) was considerably lower than the hydrogen concentration threshold necessary for hydride phase formation in Ti-α [21]. This observation confirmed that the diffused hydrogen exists solely as solute hydrogen.

(1) $$c\left(x,t\right)=c_{0}+c_{s}(1-\mathrm{erf}\left(\frac{x}{2\sqrt{Dt}}\right))$$

**Figure 3:**
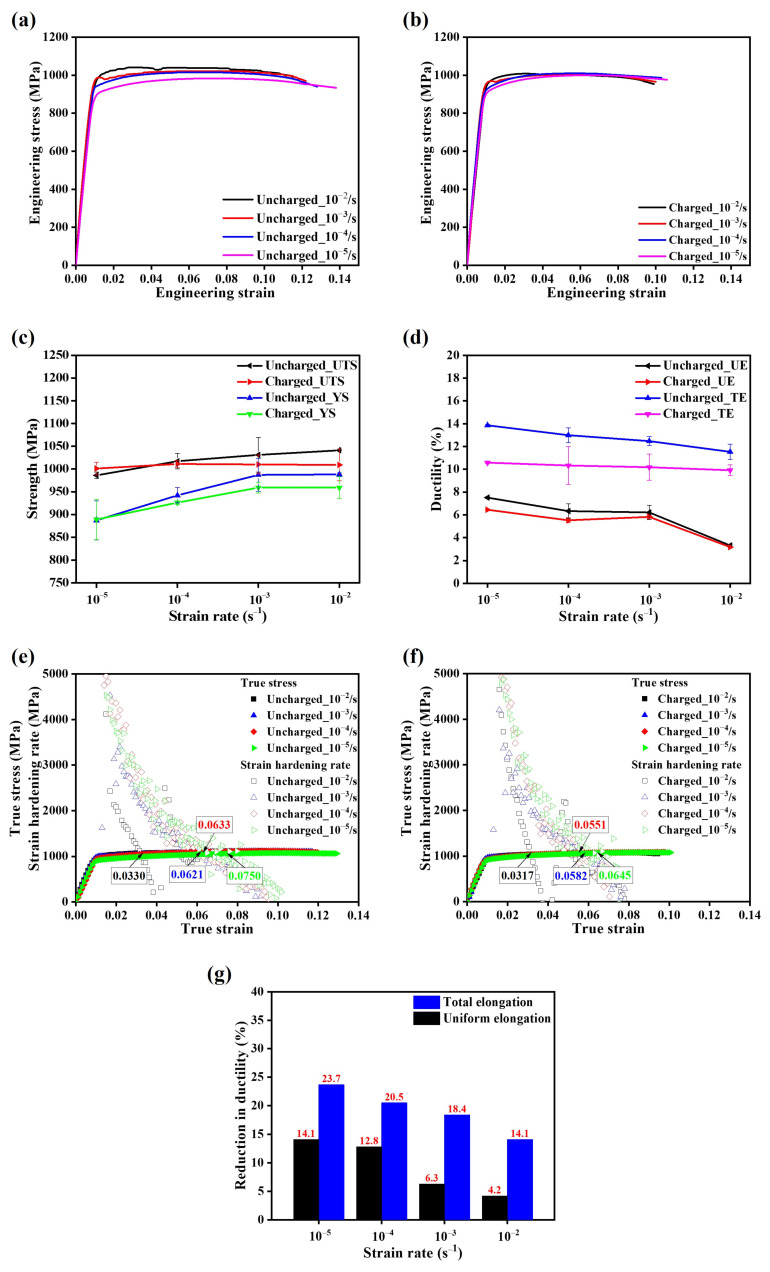
Comparison of mechanical properties of the hydrogen-charged and uncharged samples at various strain rates during tensile tests: (**a**,**b**) engineering stress–strain curves, (**c**) changes in strength vs. strain rate, (**d**) changes in ductility vs. strain rate, (**e**,**f**) true stress and strain-hardening rate vs. true strain, and (**g**) reduction in ductility vs. strain rate.

Figure 3 shows the mechanical properties of both hydrogen-charged and uncharged samples at various strain rates. The mechanical strengths of uncharged samples demonstrated a positive correlation with the increasing strain rates [34,35,36], as revealed by the widening ranges of ultimate tensile strength (UTS; 986–1041 MPa) and yield strength (YS; 887–988 MPa) in Figure 3c. In contrast, there was a gradual decrease in uniform elongation (UE) from 7.5% to 3.3%, while the total elongation (TE) decreased from 13.9% to 11.5% for uncharged samples (Figure 3d) [36]. In the hydrogen-charged samples, the correlation between mechanical properties and strain rates was inconsistent. The UTS and TE exhibited relatively small variations with the increase in strain rates, as evidenced by a slight fluctuation in UTS from 1001 MPa to 1011 MPa and a slight decrease in TE from 10.58% to 9.91%. On the other hand, the hydrogen-charged sample revealed high strain-rate dependency on YS and UE, compared to that on UTS and TE. In particular, a broadening of YS (889–959 MPa) was found with an increase in strain rates, whereas UE fell from 6.45% to 3.17%.

To investigate the variations in strengthening mechanisms with the presence of solute hydrogen, the strain-hardening behavior of both hydrogen-charged and uncharged samples was examined. Figure 3e,f demonstrate the relationship between true stress (σ_t_) and true strain (ε_t_), along with the corresponding changes in the strain-hardening rate (dσ_t_/dε_t_). The points where the strain-hardening behavior aligned with Considére’s criterion (σ_t_ = dσ_t_/dε_t_), referred to as necking points, are indicated by arrows. In the uncharged samples, the slopes of the dσ_t_/dε_t_ curves increased with the increasing strain rate. The introduction of solute hydrogen significantly influenced the strain-hardening behavior, as evidenced by the steeper slopes observed in the charged samples and the resultant reduction in necking point values. Furthermore, the impact of solute hydrogen on the strain-hardening behavior varied with the strain rates. In the high strain rate regime (10^−2^/s to 10^−3^/s), the slope of the dσ_t_/dε_t_ curves demonstrated a slight increase. Conversely, within the lower strain rate range (10^−4^/s to 10^−5^/s), the role of solute hydrogen on microstructural deformation became more pronounced, resulting in sharper slopes. Remarkably, the necking point of the hydrogen-charged sample tested at the strain rate of 10^−4^/s was even smaller than that observed for the hydrogen-charged sample tested at the strain rate of 10^−3^/s, highlighting the heightened manifestation of hydrogen embrittlement behavior at lower strain rates.

The presence of solute hydrogen in alloys has been reported to lead to two contrasting effects: strengthening due to the pinning effect and softening due to enhanced dislocation mobility [37,38,39]. Specifically, solute hydrogen can act as a solute in alloys, potentially leading to solid solution strengthening by pinning dislocations (pinning effect) [37]. However, hydrogen can also promote dislocation mobility by enhancing the mobility of mobile dislocations and reducing the elastic shielding of dislocation–defect interactions [38,39], resulting in the softening effect. These two competing effects of hydrogen ultimately lead to the predominant effect. Hong et al. [40] demonstrated that hydrogen inhibits dislocation movement at a higher strain rate (0.5/s). This suggests that under our strain rate conditions, solute hydrogen could be transported by mobile dislocations, thereby increasing the elastic shielding effect, and reducing the intensity of dislocation–dislocation interactions [14]. In this study, due to the low solute hydrogen content and its distribution mainly at grain boundaries, the softening effect is believed to be the primary influence on the Ti64 alloy, as evidenced by the reduction in the slope of the strain-hardening rate and necking points of the hydrogen-charged samples.

The hydrogen embrittlement susceptibility of an alloy is commonly measured by the decrease in ductility. While many previous studies [10,17,41,42] have predominantly focused on the reduction in TE as an indicator of HE behavior, this study delved into both UE and TE to explore the impact of solute hydrogen on ductility. The ratio 1 − X_c_/X_u_ (where X_c_ and X_u_ denote ductility values for the hydrogen-charged and uncharged samples, respectively) was calculated to assess the reduction in ductility (Figure 3g). As strain rates increased, the reduction in UE displayed a downward trend from 14.1% to 4.2%. A similar pattern was observed for the decrease in TE, specifically declining from 23.7% to 14.1%. Consequently, the sensitivity to HE gradually diminished as the strain rates increased.

**Figure 4:**
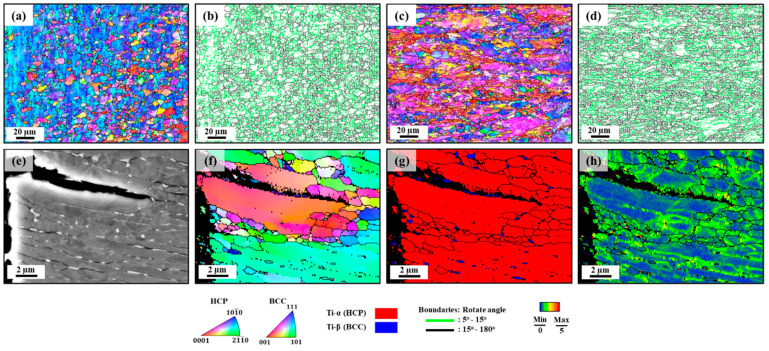
Microstructural analysis of the RD-TD plane of uncharged samples after tensile tests at different strain rates: IPF and rotation angle boundary maps at (**a**,**b**) 10^−2^/s and (**c**,**d**) 10^−5^/s. Microstructural analysis of the RD-TD plane of hydrogen-charged samples after tensile tests at a strain rate of 10^−5^/s: (**e**) ECCI, (**f**) IPF map, (**g**) phase map, and (**h**) KAM, showing hydrogen-assisted cracking at the secondary crack on the cross-section (below ~1 mm from the fracture surface).

The effects of strain rates and solute hydrogen were separately investigated to simplify the analysis. The microstructural impact with different strain rates was analyzed on uncharged samples tested at strain rates of 10^−2^ and 10^−5^/s (Figure 4a–d). Table 2 presents a summary of total boundary length/area values, providing clarity on the degree of microstructural deformation. These values were determined by calculating the ratio of the combined lengths of low-angle grain boundaries (LAGBs) and high-angle grain boundaries (HAGBs) to the total area covered by EBSD tokens. At the high strain rate (HSR) of 10^−2^/s, the insufficient time for deformation led to a fractured microstructure that was almost identical to the initial state (Figure 1b). Conversely, for the tensile test at the LSR of 10^−5^/s, some changes in the microstructure were observed (Figure 4c,d). During tensile deformation, three successive transitions could be observed: dislocation generation, LAGBs’ formation, and HAGBs’ formation [43]. Initially, plastic deformation led to the generation of dislocations. As high-density dislocations were produced in deformed grains, the tangled dislocations rearranged and released energy, leading to the formation of LAGBs. Subsequently, dislocations transitioned into stable LAGBs, and finally, the stress-driven motion of LAGBs resulted in the formation of HAGBs. The transition from dislocations to LAGBs is reported to take approximately 400 ps under stain-free conditions, and LAGBs’ migration usually involves several nanoseconds [44,45]. However, the transition from LAGBs to HAGBs requires a longer duration. In the HSR condition, a relatively large fraction of dislocations was formed, and the transition from dislocations to LAGBs occurred rapidly due to the short duration of this transition. However, the overall deformation time was insufficient to complete the transition of dislocations from LAGBs to HAGBs. On the other hand, in the LSR condition, despite the initial dislocation density potentially being smaller than that in HSR conditions, the process unfolded over an extended period, allowing continuous generation of dislocations. This provided ample time for the transition of dislocations from LAGBs to HAGBs, resulting in the eventual attainment of a relatively large fraction of HAGBs. Consequently, the short time of HSR may be insufficient for the complete transition from LAGBs to HAGBs, whereas the LSR provides ample time for the sequential evolution from dislocations to LAGBs, followed by HAGBs. Indeed, the prolonged LSR allowed for greater microstructural deformation, resulting in a higher fraction of HAGBs in the fractured samples, while the fraction of LAGBs remained similar in both cases, as summarized in Table 2. Furthermore, the IPF maps suggest a crucial role of the strain rate in shaping grain morphology and changing crystallographic orientations. Particularly, the microstructure subjected to the HSR displayed equiaxed grains, while the microstructure deformed at the LSR exhibited elongated grains. Additionally, the orientation of these elongated grains was found to be closely aligned with basal orientations.

The influence of solute hydrogen can be examined through hydrogen-assisted cracking at the secondary cracks observed near the fracture surface of the hydrogen-charged sample tested at the strain rate of 10^−5^/s (Figure 4e–h). It is crucial to emphasize that the hydrogen-charged sample only exhibited secondary cracks, which propagated along the α-phase matrix grain boundaries or the α/β interface, leading to intergranular fracture [20,46]. HAGBs, which are traditionally recognized as hydrogen traps [47,48], serve as optimal pathways to accelerate the diffusion of solute hydrogen, creating short-circuit routes for hydrogen accumulation. Crack propagation along the α/β interface can be explained based on the difference in the diffusion rate and hydrogen diffusivity between the hexagonally close-packed α phase and the body-centered cubic β phase in the alloy. At room temperature, the diffusion rate of solute hydrogen in the α phase ranged between 3.26 × 10^−17^ m^2^/s and 1.05 × 10^−15^ m^2^/s, while the diffusion rate fluctuated from 2.17 × 10^−12^ m^2^/s to 2.32 × 10^−11^ m^2^/s in the β phase [22,49]. Additionally, the reported hydrogen solubility of the β phase (20.6 × 10^3^ wppm) was higher than that of the α phase (2 × 10^3^ wppm) [21]. Consequently, the α/β interface serves as a hydrogen trap, promoting crack propagation. Therefore, the ease of crack nucleation and propagation accounts for the reduction in both uniform and total elongation in the hydrogen-charged samples.

**Figure 5:**
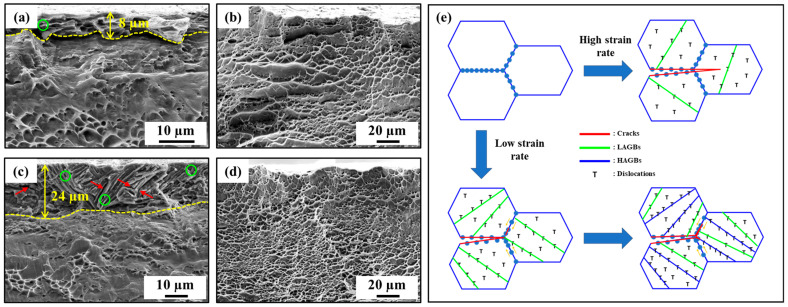
Fractography of the hydrogen-charged and uncharged samples at (**a**,**b**) 10^−2^/s and (**c**,**d**) 10^−5^/s, and (**e**) schematic illustration of the combined effects of solute hydrogen and strain rates. Green circles and red and orange arrows indicate nano/micro-voids, micro-cracks, and diffusion directions of solute hydrogen, respectively, for the hydrogen-charged samples.

Figure 5a–d show the fractography of hydrogen-charged and uncharged samples tested at strain rates of 10^−2^ and 10^−5^/s. The uncharged samples had a fully ductile fracture mode, and the HSR-tested sample displayed larger dimples than the LSR-tested sample. On the other hand, both hydrogen-charged samples revealed a hydrogen-affected layer (Figure 5a,c). In this layer, the existence of nano/micro-voids is evidence of solute hydrogen accumulation under loading. Notably, micro-cracks were found within the hydrogen-affected layer of the hydrogen-charged sample tested at the LSR (Figure 5c) but were absent in the hydrogen-charged sample tested at the HSR (Figure 5a), highlighting the influence of the strain rate. We examined the hydrogen-damaged region for the samples with HSR and LSR. In the HSR condition, the brittle layer was found to be approximately 8 µm, whereas it extended to 24 µm in the LSR condition. Indeed, the sufficient time for the tensile test under the LSR promoted solute hydrogen diffusion and accumulation, aiding hydrogen-associated cracking.

A schematic illustration of the combined effects of solute hydrogen and strain rates on HE is shown in Figure 5e. After hydrogen-charging, all samples had the same amount of solute hydrogen. However, the interaction of solute hydrogen and microstructure can vary with different strain rates. Compared to the HSR condition, the LSR condition provided extended deformation periods, fostering more extensive microstructure development. The diffused solute hydrogen layer can be characterized into two regions: a region with a high solute hydrogen concentration near the surface and the remaining portion with a low solute hydrogen concentration underneath. In the high solute hydrogen concentration region, hydrogen can easily diffuse and accumulate, promoting hydrogen-assisted intergranular cracking for both HSR and LSR conditions. In the remaining region, various reactions between solute hydrogen and microstructure may take place depending on the strain rate. Particularly, the microstructural deformation rate under the HSR conditions may exceed the diffusion and accumulation rates of solute hydrogen. This suggests that the influence of hydrogen-assisted intergranular cracking was minimal, and the fracture mechanism shifted toward a trans-granular fracture. The slow microstructural deformation rate and the abundance of HAGBs in the LSR-tested sample facilitated the diffusion and accumulation of solute hydrogen, thereby sustaining hydrogen-assisted intergranular cracking. Additionally, the generation of micro/nano-voids and micro-cracks was subject to the influence of strain rate and solute hydrogen, thereby impacting hydrogen embrittlement (HE) behavior. The accumulation of micro/nano-voids acted as structural vulnerabilities, facilitating the propagation of cracks. Furthermore, the LSR-tested sample offered favorable conditions for the formation of these voids. Elevated solute hydrogen concentrations in specific locations can induce greater stress concentrations, thereby promoting the initiation of micro-cracks. Consequently, with the increased prevalence of HAGBs and formation of both micro/nano-voids and micro-cracks under the LSR conditions, crack initiation and subsequent intergranular crack propagation would be heightened. These findings suggest enhanced susceptibility to HE with a decreasing strain rate. As a result, the effect of the LSR expanded to the brittle region damaged by solute hydrogen, leading to a substantial reduction in both UE and TE, as illustrated in Figure 3e.

## 4. Conclusions

This study investigated the combined impact of solute hydrogen and strain rate on hydrogen embrittlement (HE) of Ti6Al4V through a series of tensile experiments conducted at varying strain rates, coupled with microstructural analyses for both hydrogen-charged and uncharged samples. The results indicated an increased susceptibility to hydrogen embrittlement as the strain rate decreased from 10^−2^/s to 10^−5^/s, which was explained based on the following conclusions:

(1) The presence of solute hydrogen induced embrittlement features, as demonstrated by the formation of nano/micro-voids, micro-cracks, and hydrogen-assisted intergranular cracking behavior. However, the extent of embrittlement varied depending on the strain rate.
(2) Strain rates played a crucial role in the microstructural evolution of Ti64. Notable changes in microstructure, particularly a significant increase in the length of high-angle grain boundaries (HAGBs), were observed at low strain rates.
(3) The enlarged, brittle, hydrogen-damaged region at lower strain rates facilitated crack initiation and intergranular crack propagation, resulting in a considerable decrease in ductility of the hydrogen-charged samples.
(4) The hydrogen-charging effect on tensile strength was not substantial, likely due to the insignificant fraction of the hydrogen-affected region compared to the cross-sectional area.

## Figures and Tables

**Table 1:** The chemical composition of Ti6Al4V.

Ti6Al4V	Al	V	Fe	O	C	N	H	Ti
wt%	5.76	3.93	0.087	0.13	0.0068	0.011	0.0019	Balance

**Table 2:** Total boundary length/area of the uncharged samples after the tensile tests using the strain rates of 10^−2^ and 10^−5^/s.

Samples	Boundary	Total Boundary Length/Area (µm^−1^)
HSR-tested sample	LAGBs	0.47
	HAGBs	0.48
LSR-tested sample	LAGBs	0.46
	HAGBs	0.57

## Data Availability

The data that support the findings of this study are available upon request from the corresponding author.
